# Second Clinical Report on Continued Fever, Based on an Analysis of Forty-Eight Cases

**Published:** 1851-11

**Authors:** Austin Flint

**Affiliations:** Prof. of the Principles and Practice of Medicine, and Clinical Medicine, in the University of Buffalo


					﻿BUFFALO MEDICAL JOURNAL
AND
MONTHLY REVIEW.
VOL. 7.	NOVEMBER, 1851.	NO. 6.
ORIGINAL COMMUNICATIONS. oX
ART. V.—Second Clinical Report on Continued Fever, based on an anal-
ysis of forty-eight cases. By Austin Flint, M. D., Prof, of the Princi-
ples and Practice of Medicine, and Clinical Medicine, in the University
of Buffalo.
(Continued from Page 298.)
SECTION EIGHTH.
Symptoms referable to the circulation. [No. for Dec., 1850, page 385.]
In most of the cases the pulse was enumerated once, and sometimes twice
on each day, during the febiile career. Occasionally this was omitted, and
in a few instances the omission occurred on three or four consecutive days.
It is to be presumed, when this wTa3 the case, that the pulse presented no
material variation from what had been previously noted. In connection with
the frequency, other characters belonging to the pulse are usually given.
Whenever any unusual characters were observed, they would undoubtedly be
embraced in the daily notes. I will first direct attention to frequency of the
pulse, irrespective of other qualities, and since all the facts pertaining to this
point may be placed in a tabular form without occupying much space, I will
again adopt this method, which was pursued in the former Report. In the
following tables every thing is omitted except the numbers expressing the
frequency of the pulse per minute at the time of the daily enumerations. A
column will be devoted to each case, and the numbers placed in consecutive
order in the same column, to denote the frequency of the pulse on successive
days during the continuance of the febrile career. When the enumerations
were made both at morning and evening, the numbers are placed side by
side, and the letters P. M. placed before the latter. Sometimes it happened
that the record of symptoms was made at evening, and not in the morning.
When this was the case, as respects the pulse, the letters P. M. are placed
before the numbers. The numbers without any prefix, denote enumerations
made in the morning, or during the forenoon. The omission to enumerate,
or to note the frequency of the pulse on some days, is expressed, in the tables,
by two ciphers. In two cases of the Typhoid type, and one case of Typhus
the enumerations are so incomplete that they are omitted. The tables, there-
fore, which are limited to the cases assigned to these two types, contain the
facts pertaining to the frequency of the pulse in twenty-five cases of Typhoid,
and in nine cases of Typhus.
o	|
3 88S88SSg£SS88Ssf,
_______________Is _____________________
£
W	06	i
i 1
u	5-
©	SB
---------------------------------------
Q.	£
> L	|
I g8S8<mjeggS3E8g£
&	"	"3	§2
£	=. RS
§	g
& H	I
= g£gggSSSSSgSgggg8388SSjes?!§££ri
■ ' ........................
g 1 sissg^l
s . il«i
O -----------------------------------------
e	.	i
5a	’	g.
§	» SS3gg7gge .
g	0	§3
§	S88
---------------------------------------
O '	??
S |»	|
§ i gSSSSEEggf
S	8*
X _______________SS3_______________________
3 L	[
B - 8sgaggs?gaggsss^i
___________________J|__________________
ilIiWipifriH i ’
© © © © ©	©
O □» © © ©	©
® s' a e a a	s	§
i 38%J8gSSSSSSS8SSgg£d
5 w	rtWW wct«w
S o
____________________________S3_______________
5	S	88SS	£
tn	U	-	E S S	|
ir	’""’	d.	Q>	P» Q. CXi	O’
g I gsssgsgsss*
<1	O	ct?
o	g-
S co
--------------------------•-------------------------
kJ -----------------------_----
O	88
SJ	O
S’	J	O
Os ®	a	-
LJ	o.	c
| S8SSS*
fe	°	S3
|	ES_______________________________
§	§	§
. I	•	s
S	a	«
B J §gssf,o
° is
E _________S§___________________________________
fe I	CD (N CO 00 © M* C*	CO	M1 © ©	*
p~~*	©O-©t*b-©t*	CNQDt'-	£»
w 3 daassas	e	dss	S
02	O. O. O. O. D. o. O<	O.	0.0.0.	CT
i SSSSSSgSSSSSSSSS*
t» o -	--- C
Q.	aS
® •
Eo
O ----------------------------------------—-—
© ^ ^	*
}x	l’ <° ®	g-
o	ri	ESS	§
m	0.0.0.	O>
W	®	2 C>’I" © S’£	2
ra 5oo85oC
£	Q -	coi
Cz	g J,
Ph	**
EtJ	————-.	—	—
-r ©
<5	CT^-fsOX	O
ci	s' a a a a	S
rC	0.0 O. 0.0.	CT
g	3	2g?883SS^
H	°	”	55
s	.__________________
W	*« >- a, ~	J’
o 0> w _□ r >.
Wm— «	2
•	c K "2	°	S
S	a	e>. -Ss?~	S
PQ	£ ce C ’E —• ♦*
<i S ass °~.2 2 u § 2 .
C_j	«	'—CO
•2 g-SS S £ e|S
G <2 d o > 0.0S rn
2®3 S SSStOig'sSE £
®	ass a	a a a-o.= «“ °- I
***	d i< i a	ci, ci. ci ® g"-~ — ® cs u*
i gg88S£3S§83S3§3SgS Asif'S g
5	"	~~----3«n-’g-g °-S s«
k o o t o co oi £
q£g§5SSSZ
S a. 5.	>•
R	4 (S s
Ct Ct Ct C*
®	©©©©GO©^"©^
K3 OO	JDCJXO'C
•U	w	e-*
5«
SS
S3	— <
33.	*“ o
02	§	=• 4	4 1
y	ct a	a c*
rt C’-OOCLt-^X'*- e
“*S Q
o
Q	SS
-------------------------------------------------------------
§ *
$	.	g
ft	®	I
t>	&	e»oooo«2
a oooooot»c
t> «	~ W geo
S	5C’
u	sg
c a	-o	-
S3	3	&■
t*	S 8	8	S
$>	”	B.C.	B.	g<
<i	S	®9'3$2«	*oo«*S
Je	»	®S5S3	®»w*’*<-e5
S?	°	C-?
F	s°
tx	s-g
S	1	ss——————.	——
M «	8	8 8 8 8 8 8 8	S g S
ZZ2	d. ci d. d. d. a. d. a.	— g_g . §.
32	s	o o o co oo e» *r t o 00 o a g ® co © o« x o © co c o» ** oo oo a » c -r » oo o* — X „ o H 3
._	S	ticci-cacct w 0» o> co co co a.-r co~S^5,e< — — co — 5 X 3 S S Js »“ « 2
%	sIiIb
o -----------------------------------------------------------------
©	Ci *>©	•
>	5	3	33	£
O - s S 8 s	S
22	•	.	■ “	cc
°*	a.	a.	c-a.	©
W	g	rrcoocae	oooo«£„-
<->	§	cS co — o-r co o	cooS — CJJ
„ o -<q
X	K
W	.25
□5	22
r_	' ——————.	-
2 22 £
O	25	%•= s xj .£ o
5 3	4 44 u>S5SH
a aa	a >» on^c*
c—1	w	©©©©©©©©T-T^te c ti ? -
~	g	3SS3SSSSfao-ag-3S’S'-3
a	^-sS^gsS
«	-
W	,	g
©	g
£Q	w	c*
Q Ci WCi © © © ©*^ X *f © © © ** © © Ci
2 i*r*f*O©OGO©C>Q0t-.Q0(DC-Oi*C:-
t-	cS	HHH	CO
O	C’?
8«
____________________________gs_______________________________
©o	*
«2	o
• X	6
•	E 2	°
■2	••	s
■”	a. a.	o*
2 aoci'r-foo'f'2
5 co c ® © co o aococS
5 ’-rt ~	ccj
goo
Sao
©
o
c
.	0)
Ox	3
er
m ci o d co cr o o o >«■* co £
fl	C1H O Cl O CTO 00 C •
Q	«S
fl”
______________________aa _____________________________
£
s
CO	~
©	s4
• to O C O Tf O erd io c
,	«« ddciddcc-^cm-.
U ri T-, T-1	rR	c
rt
£ th
£
o
c
t*	5
.	_	°*
J§	m	OOWOoJf
I&5	fl	<M CI v. OiO.C'. <H
<1	gc
O ________________________gjC__________________
S"5 *-ji fl >1
S-	■“	« S o o o
sB	tea —-=xs a
E, o'	„•'S’"* >■ - S ~
nS	©	'o	'S. 9*
o « =£eo
£5g.iic£
»_	.« 2 Q o o
HH	«	~'S,Q>>S-.fl3
S	q	®SS2SSfe o «■§ "‘■E*
(J	o	A or=	a
hh	rc	z o	z>	,
b~)	o	9 o	c	2 o ci
h-'	*5	a t	o	c —< ci
f>-< « P.W <Z>*5 V4
g	jFii
S	es sf
y J,	O.B. ftfl>
g «	5§2S8£
g	g .
M	®2
w. ________________*r._____________________________________.
W	o a q.73 *r ,g £ a >»
□	«.S £0 Ja £ o * o
g «	** S
■*H	<D	rtO'e«°**OxX CT
A	“	-*•-«cdo-rco-’T J£,-o:rcoo x o £o	E
"	& OOr-KOOOO CD.CC ©O^TfvO'2—l OD O G &
Q m H H ri r’ ri H	ri H H H 2	-w CC *r*
5 -3	. £j
“gHh^gjg
a a a w £ c c 2 -
o o Jr a ►*
s C 2 o o
zc0 | o §
01	-^® = ? a
; q	js	q h ©•■H FT
w	co o co o o rt»o co cioo^r cd o o c© -* er ci d o *5 o-r
cs	©d©o©o©oao©o^dHtt~< — a-d®3 *■» C*-> £ <ir®
Q	r-^ _< r-< h -h M r- rl rH rH rH TH rl YH a ~	» a ^“7
~ ©^ C £co
© S © .2 w © o
___________________________________q 8<SggJgS
'.	is	£
E 2	|
'"'	fl. d.	er
m 00 o o o <N e •* ir~.E _J
S St — <=> o os <x> oo te-sa-^”
° -------- g£
<5 co
*5 o
On following down the columns of numbers it will be perceived that the
pulse presents, frequently, considerable variations in the same case during the
febrile career. These variations, within certain limits, appear to be due to
fluctuations incident to the progress of the disease. A striking increase in
the frequency of the pulse, unless of transient duration, and occasioned by
extraneous influences, usually accompanies some important event, which will
be likely to affect the issue of the disease. The events, in the present collec-
tion of cases, observed to be thus signalized, are the development of pneu-
monitis, e. g., case No. 3 (T’ypfozs); the occurrence of perforation of intestine,
e. g., case No. 10 (Typhoid); of a condition tending to, or eventuating in
apoplectic coma, e. g., case No. 5 (Typhus)’, a sudden change ending in
death by apnoea, e. g., case Nos. 21 and 17 (Typhoid).
A great increase in the frequency of the pulse is undoubtedly of bad omen
in Continued Fever; but it is by no means a fatal sign. In several of the
cases, it may be observed, in which the termination was in recovery, the
pulse exhibited very marked accelerations. It rose, for example, to 140 and
144 in case, No. 17 (Typhoid); to 140 and 146 in case, No. 22 (Typhoid),
and to 140 in case 24 (Typhoid). The instances in which, among the cases
ending in recovery, the pulse attained a high maximum were more numerous in
the Typhoid group. The highest number which the pulse reached in the Ty-
phus group, in the cases ending in recovery, was 140, and the highest number
after this was 128. The reverse of this was true of the first collection of cases.
The average mean, however, in frequency, is greater in the cases of
Typhus than of Typhoid., as the following comparison shows:
Average mean in the Typhus cases, 110 5-9.
Average mean in the Typhoid cases, 95 15 -27.
These results, as respects the disparity disclosed by comparison of the two
types, correspond with those developed by the first analysis. The latter gave,
as the average mean of the Typhus cases, excluding those which proved
fatal, 105 4 — 9; and of the Typhoid cases, 95 7 - 13. Excluding, in the
present collection, the fatal cases, and the average mean of the Typhus cases
is 108 1-5; and of the Typhoid cases, 91 6-21. Thus, in both analyses
the average mean frequency of the pulse is considerably higher in the cases
of Typhus, than in those of Typhoid. In the present collection, however,
the average mean frequency is somewhat higher in Typhus, and lower in
Typhoid, than in the cases before analysed. The average mean frequency is
higher in the cases which proved fatal, than in those ending in recovery.
In the fatal Typhus cases, it is, 113 1-2; in the fatal Typhoid cases, it is,
110 1-2, the disparity in the two types being still apparent.
The lowest mean exhibited in the tables, is 69 1-2, case No. 13 (Ty-
phoid). This is below the average frequency of the pulse in health. In
this case the patient entered the hospital on the day after taking to the bed,
and the fourth day after the commencement of the access. The diagnosis was
sufficiently clear, although the eruption was not developed. The disease was of
a very mild grade, convalescence being pronounced on the seventh day after tak-
ing to the bed. In this case a relapsing febrile attack occurred, and the pulse
then rose to 128. I am unable to say whether the pulse in this patient, in
health, was less frequent than the normal average. Cases of Continued Fever
presenting so low a mean in the frequency of the pulse, must be very rare.
The lowest mean next to that in the case just referred to, is 79, case No.
14 (Typhoid). In this case the pulse on one day was 128. The case was
not one of very mild grade. It was characterized by notable acceleration of
the respirations. Convalescence was pronounced on the twenty-first day
after taking to the bed.
As a general remark, it is undoubtedly true, that the mean frequency of
the pulse is a pretty good criterion of the severity of grade of the disease,
this being proportionate to the degree of acceleration. The rule, however,
is not without exceptions.
A reduction considerably below the normal standard, at or near the period
of convalescence, appears in several cases in the present collection. In case
No. 9 (Typhoid), it fell to 60; in case No. 13 (Do.), to 64; in case No. 14
(Do.), to 64; in case No. 24 (Do.), to 42. The same fact was noticed in the
former analysis. On the other hand, in several cases, at the time of conva-
lescence, the pulse was more frequent than in health. The latter fact has
been remarked upon by several observers, but the former I do not remember
to have seen noticed by writers.
It will be noticed, on reference to the tables, that the enumerations of the
pulse often disclose variations in frequency on the same day. In some of these
cases the greater relative frequency was in the morning, and in other cases at
evening. Other cases again show, on some days, greater relative frequency in.
the morning, and on other days, at evening. In so far as the pulse is an in-
dex by which to estimate febrile exacerbations, these variations show the
absence of any uniformity or regularity in their recurrence. It is certain that
diurnal exacerbations during the progress of Continued Fever, are not nearly so
often present as one is led to suppose from the current literature of the subject-
As respects the qualities of the pulse, irrespective of frequency, it will suf-
fice to give a few brief statements. It was usually either moderately or con-
siderably developed; that is, the artery appeared to have either moderate or
considerable volume. Sometimes it was small, and at the same time feeble.
If tolerably developed, the impulse communicated to the touch denoted a
kind of smartness in the systolic contraction, but it was almost invariably
compressible. It was never hard. In a few cases it was noted as vibratory,
or thrilling. If it became very frequent, it was uniformly quite soft or feeble.
To trace the connections of these variations with other symptoms in the dif-
ferent cases, would require not a small degree of labor, which it is not proba-
ble would yield results commensurately valuable. In general, it may be said,
that in Continued Fever, the circulation presents a morbid activity, not, how-
ever, involving an increase of power in the motive agencies by which the
circulation is carried on. The distinction intended to be expressed by these
terms has, theoretically, an important bearing on the subject of therapeutics.
Congestion of the capillary vessels properly enough belongs in this section,
but the facts pertaining to this subject which the analysis discloses, have been
already sufficiently considered in connection with the symptoms referable to the
general aspect, section third. The presence of capillary congestion in a large
majority of the cases of Continued Fever, is evidence that the circulation, al-
though unduly excited, is carried on less efficiently than in the state of health.
SECTION NINTH.
Symptoms (exclusive of eruptions') referable to the skin. [No. for Dec.,
1850, page 394.]
During the progress of Continued Fever, the skin generally presents a
series of changes a3 respects temperature, dryness, moisture, and sweating.
The facts pertaining to the order of succession of these changes in individual
cases, form an interesting portion of the natural history of the disease. In
the first Report a few selected cases were given illustrative of the diurnal
succession of this class of symptoms during the career of the fever. As it
will not occupy much space, I will embrace in the present Report, in a tabu-
lar form, a succinct statement of the condition of the skin (irrespective of
eruptions) in twenty four cases of Typhoid, and in eight cases of Typhus,
the histories of which are pretty complete in this particular. In the tables
which follow, the cases are arranged in parallel columns, as in those exhibit-
ing the pulse; and the symptoms, which were daily recorded, are arranged
in each column in the order of their succession, commencing with the time
the patients entered the hospital, and ending with the termination of the dis-
ease either in death, or the stage of convalescence. The days on which no
record of the condition of the skin was made, are represented, as before, by
two ciphers. Each column, thus, contains the facts pertaining to the skin
which were noted in the history of the case to which the column is devoted.
f iffmi_________________
! - J ill Jl'
£ . £ °? -“S5SS5
fc	:iHi ii
O'	a	SS
I it U list,j|ls is i
£ i"H «*!	»t §
B	Illi MEIilll
§ t iiii^
B Uli___________________
“ n 1 Hi
§ i ls==1
z s c h a
S’	« s S
S	£ £££_______________
i 7 Ah I
|	ili-II I____________
I tu fl Hl
S t illWIUHU
« ii=i H iii
’«Jim
7 Vsililiiis ijb
. i p sis Hhfh
as m	^g_______
I?t s.l 1.
s W Si I
=	1U1
££ £ £
° i T~
5	. 6f
C 12 d ~S
g h
H I ii
£ " fJisiifiifiiiiiiiii Hlhi
s, g a a a a a aaa as asa_
| MiH
3	6 >.1F=^
g r |fi= f
LTtlB
i * l>IjjaIij;l
H	fa xs? x ?
I t !l a>f
e Mi J J
° iHUin
Hm:ip
-------------.----------
. .	i
w >» .	. o
• o
5 E	■eg
o	=
.	-	.’S -	c -
co	£	&c	22
LxJ	cd	x-. cd	cd cd
co	£	££	££
•< .—----------------------------------------------------
°	§	fcS	3	>	>
O\’4’S°-5x5	O t o
*3	|	2	=5 2	-g£?	g	2?	®	6
?	«	ST3	5	y.	s	b	2	Z
-	E	’E S	o o"c o o O o o ° ® O o ° ° © ° ° °a sZ	cS1®	Z	2 2	q
£ gj « g" aZEaaQaaaaaaQaaaaaga« ga-c saa.-s
b	6 E^EE^ Ik	g'EEE "4k i§
~	£ ££ «£	b £££	S=£
S ------—--------:—z— -----------------------------------
©	as	BBS
El	""	.	.2	2«
k1	•	t>*X u? u? 3> 75
r	8	ciEi
g 6	§©S =’5 = = ?^E
1	£ eI^Iee-Sse^
S	Be fc S . Be
3	2 • 2 . >■ . 2
-go ® “•■S S'©
rt	<K>	E^E^-o-oE
W	.	■c'=-a = ;-	6
“	5	gSg^^Sg	a
S	6	g8E§£gES
M Z 52>? 6 ^222 2
F	£££ Q
O
H	§•	3
M	-So
S	§	-s s
*	X	§dC°
•<	o	b g s a
Pi	£	"<S a
pq	8-- £
Qj	1
™	&ZS	*
CO	o rt rt	Q
<<	■£ £ sS	>•£
O 2 E £ c j10 8
E-1	2'a^2’E’2o
£q 2 s S « g ee E a
* ee^-See
c B g £ e a
e -------------------------------------------------------
£	:§•"«	.«'£	£fg .£	I
~ 2-q "	>.o 8	2oia ®2
g	aS '056	F	I!* '11
td	5 st oofccsoU 9 x 5 o. >»3S - o
Ed X E -2 o Q c « J- £ °®Srtdu2XQ
d 2	rt rt <2 • tr	W 'C ’S.	rt	ce	T5	rt
Eq. E -g	p. E E a .	8	E •© -c	E S
« ” § ciE	" ® 0.5 2	n	a « g	» "
H	££	&	££ ' 8	££	££
31	—----------------------------------------------------
W	s §	" • & 8 . B:	U
2	o «s	3 ^.2 2 . £2	.2
H	E	- g ol ©•’c «	2
£q —p	§ g a 8 wts E	8
OBx3SB’an3rt8lSd’2
0	ti£ - rt «3* fl rt
E^§EE£§fs S
2	« « § ©-tS-S 6 >S
3	O	>	>
.	. 1 11 is I
IWUj
.. t « H! I
i jgw*____________________
I I b
g =- 88I?I^
& iff 5
S s'- 8
s ----------------•-------—
* •» *
s J «U
g 5, «huu
s 4 ill
g ___*a» ..,______________
§ . J til
« 4 ps.:oii
I 1 l"Ul__________________
I J rfpi ii
g :■ 88ii»i<y tu4
g 4 fiHh =h
ass s Isa
S	fe-fe > i x >*>&•
s 1	111 Hit Ml
i ssslsppH
a m i ihw*
:-:
« |4sli
I ?lh!
The data contained in the foregoing tables, show that free perspiration, or
sweating, occurred in fourteen, of the twenty-four cases of Typhoid, and in
three, of the eight cases of Typhus. In the first collection, this symptom was
present in fourteen, of twenty-seven cases of Typhoid, and in five, of twelve
cases of Typhus. The results of the two analyses thus do not greatly differ.
Of the fourteen cases of Typhoid characterized by sweating, it is noted to
have occurred but onee in the histories of seven; twice in four cases; three
times in one case; seven times in one case, and six times in one case.
Of the three cases of Typhus, characterized by sweating, it is noted to
have occurred once in the history of one case, twice in one case, and three
times in one case.
It is a curious coincidence mostly of passing remark, that of the fourteen
Typhoid cases in the first, and of the fourteen Typhoid cases in the second
collection, characterized by sweating, in precisely the same number, viz.,
seven, this event is noted to have occurred but once in each case.
The sweating, in degree, as before, differed at different times, as also in
duration. Frequently it was quite profuse, drops standing more especially
on the face. It was observed, as before, also, to occur or commence oftener
at night than during the day.
Moisture of the surface is noted to have been present, more or less, as
respects the number of days, in seventeen cases of Typhoid, and in four cases
of Typhus. This condition obtained in a larger number of cases in the pre-
sent collection than in the cases before analyzed. The number of times in
which it was noted in the cases severally need not be enumerated. It will
be seen, on glancing at the tables, that, of the cases in which it was present,
it was noted generally several times, alternating sometimes with free perspir-
ation, and sometimes with dryness of the skin.
The two Typhoid cases in which profuse perspiration occurred the great-
est number of times, (viz., in one six, and in the other seven times), claim
particular notice. Case No. 6, in which it occurred seven times, was of
medium severity, convalescence being pronounced on the nineteenth day
after admission. The convalescence was retarded by the complication of
pneumonitis.
Case No. 18, in which it occurred six times, was also complicated with
pneumonitis. In other respects the disease was not unusually severe, and
convalescence was pronounced on the sixteenth day. Thus both cases ended
favorably.
Directing the attention now to the fatal cases, of the seven Typhoid cases
proving fatal, free perspiration occurred in three, and moisture of the surface
in two. Of the four cases of Typhus ending fatally, free perspiration occur-
red in two, and moisture of the surface in two. These results accord with
those developed by the first analysis, and confirm the conclusion that the
occurrence of sweating, or moisture, has no special bearing on the prognosis.
The facts apparent on reference to the tables, confirm another conclusion
deduced from the results of the analysis, viz., moisture, and free perspiration
do not, as is frequently imagined, exert a manifest salutary influence on the
progress or issue of the disease. On reference to the tables it will be found
that these conditions of the skin were observed at different periods of the
febrile career, in the large proportion of instances not preceding, by a short
space of time, the date of convalescence. Moreover, these appearances were
observed in nearly one half of the cases which ended fatally. We are not
warranted, then, in predicating expectations of speedy convalescence, or recov-
ery, upon either of these symptoms disconnected from other circumstances;
nor do these results afford any grounds for supposing that to induce moisture
or sweating by therapeutical means will be likely to prove beneficial.
The perspiration in Continued Fever has been said to emit a peculiar odor
characteristic of the disease. I have directed some attention to this point,
but I could never satisfy myself of the existence of such a symptom. The
Sisters at the Hospital, and some of the Students, have frequently assured
me that they were sensible of a distinctive odor arising from the bodies of
fever patients under my charge, but I have always failed to verify, to my
own satisfaction, this diagnostic. It would be assuming too much to distrust
the ability of others to recognize the disease by the olfactory sense, and the
probable, as well as the more modest inference is, that the ill success which
has attended my efforts, is due to a want of sufficient acuteness to appreciate
impressions received from this source.
The temperature of the surface, as estimated by the touch, varied consider-
ably in most of the cases on different days during the progress of the disease.
Coolness was seldom observed, but frequently the heat did not appear to be
above the normal standard. In eight only of the twenty-four Typhoid cases,
and in two of Typhus the skin on one or more days is noted as hot; and in
five Typhoid cases, and one case of Typhus it is noted as rather hot. In
the majority of instances the elevation of temperature thus designated, was
present in the early part of the disease. A burning heat is noted in only one
case — a case of the Typhoid type. The description by some writers give
the impression that an acrid heat (color mordicans) is frequently present in
both Typhoid and Typhus fever, but more especially in the latter, of which
it is sometimes mentioned as a characteristic feature. My observations do
not show it to be a frequent symptom in cases of either type, nor that is
distinctive of either. On this point the results of the two analyses concur.
Finally, it will be perceived, on reference to the tables, that a warm and
mellow state of the skin, in other words a normal condition, obtains, more or
less, during the febrile career, in a certain proportion of cases. It might be
imagined that this would not occur except when the disease was remarkably
mild, or only in connection with other symptoms of a favorable character.
Such, however, does not appear to be the fact. The skin was at times nor-
mal in cases of severity, indeed in some of those which ended fatally; and
when the symptoms in general did not denote improvement.
In view of the foregoing facts, it does not appear that the skin in Con-
tinued Fever, so far as it has been considered in this section, affords symp-
toms which possess much special significance or importance: that is to say,
we are not warranted in deducing from the different conditions of this struc-
ture, as respects sweating, moisture, or temperature, any positive inferences
concerning the tendencies and probable issue of the disease, or therapeutical
indications. A survey of the results developed by the present and former
analysis seems to lead to this conclusion, which, it is very probable, does not
accord with the notions entertained by some practitioners. The investiga-
tion by means of enumerations, and comparisons of the phenomena in a series
of recorded cases, not infrequently compels us to abandon, or modify pre-
conceived ideas based on speculation, or imperfect generalizations which
exert more or less influence upon the exercise of our art. So far, the effect
of such an investigation is negative in its character, but the advantage is not
less positive than if an important practical principle had been developed. To
disprove an erroneous opinion is often scarcely less useful than to discover a
new truth; for, in the practice of medicine, it is an object not less to avoid
acting under false views, than to adopt and apply those which science may
establish.
Gangrene. Gangrenous ulceration of the skio is noted in the histories of
two cases, one of the Typhoid, and the other of Doubtful type. In the for-
mer case it was observed on the third day after admission, the latter being
several days after the commencement of the fever. The case terminated
fatally the day after the gangrene was discovered. It was situated on the
left nates. In the other case it was discovered about the time of convales-
cence, which was on the eighteenth day after taking to the bed. This case
was severe. The patient had considerable embonpoint. The eschar was not
large, and rapidly headed after convalescence was established.
Erysipelas did not occur in any of the cases in this collection.
SECTION TENTH.
Symptoms referable to the Genito-urinary System.
A daily record of the results of a careful analysis of the urine during the
progress of Continued Fever, in an extended series of cases, would form a
valuable contribution to the natural history of this disease. Such an accu ■
mulation of observations, taken in connection with other classes of symptoms,
would offer an interesting field for analytical exploration. The task of col-
lecting the data for an investigation of this kind, so far as I know, is yet to
be performed. It is, of course, impossible to foresee the facts and relations
which would be disclosed. They might prove to be both curious and important.
But even if nothing striking or useful should be developed, an end of not a
little consequence would be attained, inasmuch as it would then be settled,
that, with our present means of pathological research, nothing is to be looked
for in that direction. The toil of cultivating the domains of science is best
rewarded, it is true, by the abundance and richness of the harvest, yet to
determine whence no such reward is to be expected, will compensate for not
a little labor by restricting future efforts within the compass of a fruitful soil.
After these remarks, I regret to state, with respect to facts pertaining to
the subject of this section, that this Report will be as meagre as the first.
When I began to record the cases embraced in the present collection, I
resolved to devote special attention to the urinary secretion. The difficulties
in the way of prosecuting the undertaking, however, soon led to its abandon-
ment, and, indeed, it was never fully entered upon. To carry out the plan
remains for a future attempt, and perhaps for those whose opportunities are
more favorable, or who are more persevering in overcoming obstacles. The
histories of the cases contain only a few events which were of a character to
attract notice. They will occupy but a brief space.
Retention of urine is noted in the histories of several cases, viz., ( Typhoid)
in one case no urine was passed for two days, when the catheter was employ-
ed, after which no further trouble arose from this source. In another case,
on the third day after admission, a resort to the catheter became necessary,
and a quart of urine was drawn off. The difficulty in this case did not recur.
Retention in a less degree, relieved without the surgical remedy, is noted in
three cases. In all these cases symptoms of nervous disorder were prominent.
The symptom is undoubtedly due, in most instances, to the blunted percep-
tions, the patient not experiencing suffering from the accumulation, until the
distension becomes so great that the power of voluntary evacuation is
impaired or lost. The importance of exploring the hypogastric region, and
ascertaining daily if urine has been discharged, is sufficiently obvious.
Retention is not noted to have occurred in any of the cases of Typhus, or
those of Doubtful type.
In several instances the discharge of urine took place in bed, either
involuntarily, or from indifference.
Menstruation occurred in one case during the progress of the disease, on
the fifth day after admission, which was several days before convalescence
was established. The case was one of mild grade.
In one case, of the Typhus type, the patient made complaint of pain in
urinating, about the time convalescence was established.
In one case, of Doubtful type, considerable difficulty and pain was
experienced in the act of micturition during a portion of the febrile career.
These are all the facts pertaining to the genito-urinary system which the
histories embrace.
(To be Continued.)
				

